# Complications maternelles précoces de la césarienne: à propos de 460 cas dans deux hôpitaux universitaires de Yaoundé, Cameroun

**DOI:** 10.11604/pamj.2015.21.265.6967

**Published:** 2015-08-07

**Authors:** Jean Dupont Kemfang Ngowa, Anny Ngassam, Jovanny Tsuala Fouogue, Junie Metogo, Alexis Medou, Jean Marie Kasia

**Affiliations:** 1 Departement de Gynécologie-Obstétrique, Faculté de Médecine et des Sciences Biomédicales, Université de Yaoundé I, Yaoundé, Cameroun; 2 Service d'Anesthésie-Réanimation, Hôpital Général de Yaoundé, Yaoundé, Cameroun

**Keywords:** Césarienne, complications, Yaoundé, Cameroun, Ccaesarean section, complications, Yaounde, Cameroon

## Abstract

**Introduction:**

La césarienne est l'une des interventions chirurgicales courantes en obstétrique. L'objectif de cette étude était de déterminer l'incidence des complications maternelles précoces de la césarienne dans deux hôpitaux universitaires de Yaoundé.

**Méthodes:**

Il s'agissait d'une analyse descriptive d'une cohorte de 460 cas de césariennes à l'Hôpital Central de Yaoundé (HCY) et à l'Hôpital Général de Yaoundé (HGY) pendant la période du 1^er^ avril au 30 septembre 2012.

**Résultats:**

Le taux de césarienne était de 19,7% dans l'ensemble de la population de l’étude, 18,64% à l'HCY et de 23,73% à l'HGY. Les indications de la césarienne étaient prophylactiques dans 191 cas (41,52%), urgentes en dehors du travail dans 67cas (14,56%) et urgentes au cours du travail dans 202 cas (43,91%). L'incidence des complications maternelles précoces était de 20,11% à l'HCY (69/343 cas), de 7,69% à l'HGY (9/117 cas) et de 16,95% dans l'ensemble (78/460 cas). Les complications hémorragiques étaient les plus fréquentes, 39(8,48%) cas dans l'ensemble, 35(10,2%) cas à l'HCY et 4(3,42%) cas à l'HGY. Tandis que les complications infectieuses étaient retrouvées dans 33(7,17%) cas dans l'ensemble, 31(9,04%) cas à l'HCY et 2(1,7%) cas à l'HGY. La différence des incidences de complications entre l'HCY et l'HGY était significative tant dans l'ensemble des morbidités (20,11% vs 7,69%; P=0,002) que pour les complications hémorragiques (10,2% vs 3,42%; P=0,02) et infectieuses (9,04% vs 1,71%; P=0,008).

**Conclusion:**

Les complications maternelles précoces de la césarienne dans notre milieu restent considérables. Les complications hémorragiques et infectieuses sont les plus fréquentes.

## Introduction

La césarienne est l'une des interventions chirurgicales les plus couramment effectuées en obstétrique et est certainement l'une des plus anciennes opérations en chirurgie [1, 2]. Son incidence est en hausse de part le monde entier [3, 4]. L'incidence de la césarienne varie d'un pays à l'autre et d'un hôpital à l'autre à l'intérieur d'un même pays [5]. Au cours des 30 dernières années, l'incidence de la césarienne a augmenté de 5% à environ 25% voire plus de 50% dans certains pays [5]. Le risque et la sécurité associés à l'accouchement par césarienne diffèrent d'un endroit à l'autre du monde [6]. Malgré les immenses progrès réalisés en technique obstétricale et en anesthésie pour offrir une meilleure sécurité materno-fœtale lors de la césarienne, les taux de complications maternelles restent élevées, mettant quelques fois en jeu le pronostic vital et le devenir obstétrical des patientes. En Afrique, quelques auteurs ont rapporté l'incidence des complications maternelles de la césarienne qui varie de 10,3% dans une étude marocaine à 40,55% dans une étude guinéenne [7–9]. L'objectif de cette étude était de déterminer l'incidence des complications maternelles précoces de la césarienne à L'Hôpital Central de Yaoundé (HCY) et à l'Hôpital Général de Yaoundé (HGY) à partir de l'analyse d'une cohorte de 460 cas de césariennes.

## Méthodes

Nous avons mené une étude de cohorte prospective descriptive sur une période de 6 mois allant du 1^er^ avril 2012 au 30 septembre 2012 dans les services de gynécologie-obstétrique de l'Hôpital Général de Yaoundé (HGY) et de l'Hôpital Central de Yaoundé (HCY) qui sont parmi les hôpitaux universitaires de référence de la ville de Yaoundé. Le service de Gynécologie-Obstétrique de l'Hôpital Général de Yaoundé a une capacité de 52 lits et réalise environ 934 accouchements par an. Les accouchements y sont réalisés par les sages-femmes et les résidents en Gynécologie/Obstétrique sous la supervision des obstétriciens. Cependant, la césarienne y est réalisée par l'obstétricien assisté par le résident de Gynécologie/Obstétrique. L'antibioprophylaxie à base de céfazoline (2 grammes après clampage du cordon et 1 gramme 6 heures plus tard) est pratiquée systématiquement sauf pour les cas de césarienne après rupture de membranes pour lesquels une antibiothérapie à base d'amoxicilline-acide clavulanique est instituée pour une durée de 7 jours. La prévention de la maladie thromboembolique par le lever précoce au premier jour postopératoire et par une héparinoprophylaxie à bas poids moléculaire pendant cinq jours y est systématiquement appliquée. A la Maternité Principale de l'Hôpital Central de Yaoundé qui a une capacité d'accueil de 66 lits et qui réalise environ 3000 accouchements par an, les accouchements sont conduits par les sages-femmes et les résidents de Gynécologie/Obstétrique et les césariennes sont très souvent réalisées par les résidents sous la supervision des obstétriciens. Dans tous les cas une couverture antibiotique est réalisée à base de Ceftriaxone (2 grammes après le clampage du cordon puis 1 grammes toutes les 12 heures pendant 48 heures et enfin 1,2 grammes d'amoxicilline-acide clavulanique toutes les 12 heures per os pendant 5 jours). La prévention de la maladie thromboembolique est assurée par le lever précoce au premier jour postopératoire et par une héparinoprophylaxie bas poids moléculaire pendant les 2 premiers jours postopératoires.

Pendant les 6 mois d’étude, nous avons enregistré un total de 460 cas de césarienne dont 343 cas à la l'Hôpital Central de Yaoundé et 117 cas à l'Hôpital Général de Yaoundé. Pour chaque cas, nous nous sommes intéressés à la mortalité et la morbidité maternelles (le décès maternel et les complications hémorragiques, traumatiques, infectieuses, thromboemboliques et anesthésiques) en per et post opératoires de césarienne survenues dans les 30 jours suivant l'opération. La complication hémorragique était considérée pour les cas présentant un saignement de plus de 1000 cc, une transfusion sanguine en per ou post opératoires ou une hystérectomie d'hémostase. Nous avons retenu comme infections post opératoires, les infections de la plaie opératoire, les endométrites, les péritonites, les infections urinaires et respiratoires. Au moyen d'une fiche technique pré-testée, les données suivantes ont été collectées auprès des participantes, dans leurs dossiers médicaux et dans le registre du bloc opératoire: l’âge, la profession, niveau d'instruction, le mode d'admission à l'hôpital (référence des formations sanitaires d’échelon inférieur ou femme suivie en prénatale à l'HGY ou à l'HCY), le caractère urgent ou électif de la césarienne, l'indication de la césarienne, la qualification du chirurgien principal (résident ou gynécologue-obstétricien), le type d'anesthésie (anesthésie générale ou rachianesthésie). Les différentes complications maternelles survenues pendant la césarienne et les trente premiers jours postopératoires ont été consignées sur la fiche technique. Les données ont été saisies et analysées au moyen des logiciels Microsoft Excel^®^ 2010 et SPSS^®^ version 13. Les fréquences et les moyennes des différentes variables ont été calculées. Nous avons comparé l'incidence des différentes complications de la césarienne dans les deux hôpitaux par le test de Chi2. Le seuil de significativité était considéré pour p < 0,05.

## Résultats

Pendant la période d’étude, 2330 accouchements ont été enregistrés dont 1840 à l'HCY avec 343 (18,64%) cas de césariennes et 493 à l'HGY avec 117 (23,73%) cas de césariennes. Au total 460 (19,74%) cas de césariennes avaient été réalisés pendant la période de cette étude.

### Caractéristiques générales de la population de l’étude

Le Tableau 1 présente les caractéristiques sociodémographiques et cliniques des participantes à l’étude. L’âge moyen des patientes était de 28,13 +/- 0,93 ans. Plus de la moitié des femmes césarisées (63,69%) étaient âgées de 30 ans ou moins. Les patientes transférées des structures de santé ayant un plateau technique moins développé que celui des deux hôpitaux universitaires (HGY et HCY) étaient significativement plus représentées dans la population de l'HCY par rapport à celle de l'HGY (57,7% vs 1,7%; P < 0.001). Dans l'ensemble de la population de l’étude, les césariennes étaient réalisées en urgence dans 58,47% des cas. Par ailleurs, les césariennes étaient réalisées par les résidents en gynécologie obstétrique dans 53,91% des cas et par les gynécologues/obstétriciens dans 46,08%. Le pourcentage des résidents en gynécologie-obstétrique comme premier opérateur dans les césariennes était significativement plus élevée à l'HCY qu’à l'HGY (71,72% contre 1,7%; P < 0,05).

**Tableau 1:** Caractéristiques générales de la population de l’étude

Variables	Population totale		HCY		HGY		p
	(N=460)		(N = 343)		(N = 117)		
Age	n	%	n	%	n	%	
≤30 ans	293	63,69	225	65,59	68	58,11	0,146
>30 ans	167	36,30	118	34,40	49	41,88	
Profession							
- Femme au foyer	185	40,21	167	48,7	18	15,4	0,000
- Emploi rémunerateur	190	41,30	121	35,27	69	58,97	
-Elève/étudiante	85	18,47	55	16,0	30	25,6	
Utérus cicatriciel	87	18,91	63	18,36	24	20,51	0,609
Pre éclampsie/Eclampsie	22	4,78	19	5,53	3	2,56	0,193
Patiente transférée d'un centre de santé	200	43,47	198	57,7	2	1,7	0,000
Indications de césarienne							
-Urgente (à l'admission)	67	14,56	56	16,32	11	9,4	0,000
- Urgente (au cours du travail)	202	43,91	153	44,60	49	41,88	
-Prophylactique	191	41,52	134	39,06	57	48,71	
Qualification de l'opérateur							
- Gynécologue-obstétricien	212	46,08	97	28,3	115	98,3	0,000
- RésidentenGynéco–Obstétrique	248	53,91	246	71,72	2	1,70	
Type d'anesthésie							
- Rachianesthésie	360	78,26	274	79,9	86	73,5	0,149
- Anesthésie générale	100	21,73	69	20,1	31	26,5	

HGY : Hôpital Général de Yaoundé ; HCY : Hôpital Central de Yaoundé

### Complications maternelles précoces des césariennes

Les complications maternelles des césariennes sont représentées dans le Tableau 2. L'incidence des complications maternelles précoces observées étaient de 7,69% (9 cas sur 117) à l'HGY, de 20,11% à l'HCY (69 cas sur 343) et de 16,95% (78 cas sur 460) dans l'ensemble de la population de l’étude. Les complications hémorragiques ont été les plus observées avec une incidence de 8,48% dans l'ensemble de l’échantillon et une incidence significativement plus élevée chez les femmes césarisées à l'HCY par rapport à celles opérées à l'HGY (10,2% contre 3,42% P=0,023). Les complications infectieuses (7,17%) étaient au second rang des complications enregistrées chez les femmes césarisées. Cependant, ces complications infectieuses étaient significativement plus fréquentes à l'HCY par rapport à l'HGY (9,04% contre 1,71%; p=0,008). L'endométrite était la complication infectieuse la plus fréquente (22 cas sur 33 soit 66,67%).

**Tableau 2:** Complications maternelles précoces des césariennes

Complications maternelles	Population totale		H CY		HGY		*p*
	(N = 460)		(N= 343)		(N= 117)		
	n	%	n	%	n	%	
Complications hémorragiques	39	8,48	35	10,2	4	3,42	0,023
Hémorragie > 1000 ml	19	4,13	18	5,24	1	0,85	
Hystérectomie d'hémostase	3	0,65	2	0,58	1	0,85	
Transfusion sanguine	13	2,82	12	3,49	1	0,85	
Hématome pariétal	4	0,86	3	0,87	1	0,85	
Complications Infectieuses	33	7,17	31	9,04	2	1,71	0,008
Endométrite	22	4,78	22	6,41	0	0,00	0,005
Infection de la paroi	8	1,73	6	1,74	2	1,70	
péritonite	2	0,43	2	0,58	0	0	
Bronchopneumonie	1	0,21	1	0,29	0	0	
Autres complications	6	1,3	3	0,87	3	2,56	NA
Complications respiratoires non Infectieuses	2	0,43	0	0	2	1,70	
Complications thromboembolique	1	0,21	0	0	1	0,85	
Arrêt cardiaque per opératoire	2	0,43	2	0,58	0	0	
Plaie vésicale	1	0,21	1	0,29	0	0	
**Total**	**78**	**16,95**	**69**	**20,11**	**9**	**7,69**	0,002

HGY : Hôpital Général de Yaoundé ; HCY : Hôpital Central de Yaoundé

## Discussion

Ce travail sur les complications maternelles de la césarienne basé sur 460 cas dans deux hôpitaux universitaires de Yaoundé a la particularité de donner un aperçu de notre pratique obstétricale. Nous avons trouvé que la fréquence des complications maternelles de la césarienne est de 16,95% dans l'ensemble de la population de l’étude et de 20,11% à l'HCY et 7,69% à L'HGY. Comparé aux données de la littérature, le taux de complications maternelles de 20,11% retrouvé à l'HCY est proche de ceux rapportés en 2004 par Häger et al en Norvège (21,4%) et par Mariko et al en 2007 au Mali (22,7%) [10, 11]. Cependant, nos résultats sont de loin inférieurs à ceux rapportés au Centre Hospitalier Universitaire Ignace Deen de Conakry (Guinée) où l'incidence des complications maternelles de la césarienne était de 40,55% [9]. Il faut noter que les complications de la césarienne dans cet hôpital Guinéen étaient dominées par l'infection qui représente 71% de toutes les complications. Cette fréquence élevée de l'infection post opératoire pourrait s'expliquer par la rupture de l'asepsie péri-opératoire très souvent observée dans les blocs opératoires en Afrique [12]. L'incidence de l'infection post opératoire moins élevée dans la présente étude que dans celle menée en Guinée serait tout simplement la conséquence d'une amélioration du niveau du plateau technique dans nos deux hôpitaux universitaires, la Maternité de l'HCY ayant bénéficié il y a quelques années d'une rénovation totale. Il découle de la littérature que les césariennes en urgence présentent environ 10 fois plus de complications (20 à 30%) que les césariennes électives (2 à 3%) [8, 13, 14]. L'incidence des complications maternelles de la césarienne plus élevée à l'HCY par rapport à l'HGY (20,11% vs 7,69%; p<0,001) pourrait s'expliquer par le pourcentage plus grand de césariennes d'urgence, de patientes transférées en urgence des centres de santé et de résidents en gynécologie comme premier opérateur dans la population d'étude à l'HCY par rapport à l'HGY (respectivement de: 60,93 contre 51,28%; 57,7% contre 1,7%; 71,62% contre 1,7%). Les complications hémorragiques de la césarienne représentent une cause importante de morbidité et de mortalité maternelles. Leur incidence est diversement appréciée selon la définition adoptée et leur moment de survenue. Ces hémorragies peuvent être dues à des facteurs généraux tels les troubles de la coagulation et de l'hémostase ou à des facteurs locaux d'origine placentaire, utérine ou secondaires à des lésions traumatiques [15–17].

Les complications hémorragiques étaient les plus fréquentes dans la présente étude et concernaient 8,48% des patientes. Cette incidence est comparable à celle retrouvée dans la littérature qui varie de 7,3 à 16,5% [7, 14]. Par ailleurs, le taux de complications hémorragiques significativement plus élevé à l'HCY qu'à l'HGY (10,2% vs 3,42%; P=0,023) pourrait s'expliquer d'une part par le fait que les césariennes étaient principalement réalisées par les résidents en Gynécologie-Obstétrique à l'HCY (71% des cas) par rapport à l'HGY (1,7% des cas) et d'autre part par la différence de niveau du plateau technique entre les deux hôpitaux. Au bloc opératoire de la Maternité de l'HCY, le bistouri électrique nécessaire pour faciliter l'hémostase au cours de la césarienne n'était pas disponible pendant la période de cette étude. Les complications infectieuses représentaient une cause majeure de morbidité chez les patientes césarisées. Elles varient dans la littérature de 6,7% dans l'étude marocaine à 29,7% dans l'étude Guinéenne [7, 9]. De nombreux travaux ont pu établir l'intérêt de l'antibioprophylaxie dans la réduction de l'incidence de ces complications infectieuses [18, 19]. Dans la présente étude, l'incidence des complications infectieuses est de 7,17% dans l'ensemble de la population de l’étude et se situe dans la tranche retrouvée dans la littérature [7, 9]. Le taux de complications infectieuses de 9,04% observé à l'HCY est proche de celui de 12,6% rapporté par Shrestha au Népal et de celui de 10% rapporté par Cissé au Sénégal [20, 21]. A l'HCY ces complications infectieuses sont plus élevées (9,04%) qu’à l'HGY (0,9%) en dépit de la pratique d'une antibiothérapie systématique de couverture de 7 jours, à cause de la rupture d'asepsie péri opératoire fréquente. Ces résultats viennent confirmer la place prépondérante du respect de l'asepsie péri-opératoire dans la prévention de l'infection post opératoire. L'incidence de l'endométrite post césarienne de 4,78% dans la présente étude est proche du taux de 5,1% rapporté dans l’étude marocaine [7]. La mortalité maternelle après césarienne n'est plus ce que l'on observait il y a longtemps, grâce aux progrès des techniques de réanimation, d'anesthésie, de l'antibiothérapie et de la transfusion sanguine. Dans l’étude Marocaine, la mortalité maternelle après césarienne est de 2,8 pour mil soit un risque 3 fois supérieur à celui de la voie basse [7]. Elle était de 1,4% dans l’étude de Cissé et al au Sénégal et de 3,45% dans l’étude guinéenne [9, 21]. Contrairement à ces auteurs, l'absence de décès maternel constatée dans notre série pourrait s'expliquer par la petite taille de notre population d’étude par rapport aux études citées ci-dessus. Cependant, il est connu que la césarienne est associée à un risque beaucoup plus élevé de décès maternel que l'accouchement par voie basse [22, 23].

## Conclusion

Il ressort des résultats de notre étude que les complications maternelles précoces de la césarienne de 16, 95% restent considérables. Ces complications sont significativement plus élevées à l'Hôpital Central de Yaoundé par rapport à l'Hôpital Général de Yaoundé. Les complications hémorragiques et infectieuses sont les plus fréquentes. Ces résultats interpellent à un renforcement des mesures d'asepsie péri-opératoire dans nos blocs opératoires afin de réduire les complications infectieuses. De même, une plus grande sensibilisation du personnel de santé à une meilleure préparation à l'accouchement en fin de grossesse permettra de réduire la fréquence des patientes transférées en urgence des centres de santé vers les hôpitaux universitaires pour une césarienne.

## References

[1] World Health Organization (1985). Appropriate technology for birth. Lancet.

[2] Kwawukume EY, Kwawukume EY, Emuveyan EE (2002). In: Caesarean section in Comprehensive Obstetrics in the Tropics.

[3] Nwosu C, Agumor K, Aboyeji AP, Ijaiya MA (2004). Outcome of caesarean section in a sub-urban secondary health care facility in Nigeria. Nig Med Pract.

[4] Stanton CK, Sara A, Holtz SA (2006). Levels and Trends in Cesarean Birth in the Developing World. Stu Fam Plan.

[5] Marc H, Alan HD, Lauren N (2007). Operative Delivery. Current Obstetrics and Gynecological Diagnosis and Treatment.

[6] Villar J, Valladares E, Wojdyla D, Zavaleta N, Carroli G, Velazco A, Shah A (2006). Caesarean delivery rates and pregnancy outcomes: the 2005 WHO global survey on maternal and perinatal health in Latin American. Lancet.

[7] Van Roosmalen J, van der Does CD (1995). Caesarean birth rates worldwide: a search for determinants. Trop Geogr Med.

[8] Hassan A, Abderrahim A, Fadila M, Noureddine M, Amine H, El Mansouri A (2001). Complications maternelles des césariennes: analyse rétrospective de 3 231 interventions à la maternité universitaire de Casablanca, Maroc. Cahiers d’études et de recherches francophones / Santé.

[9] Ugwu EOV, Obioha KCE, Okezie OA, Ugwu AO (2011). A Five-year Survey of Caesarean Delivery at a Nigerian Tertiary Hospital. Ann Med Health Sci Res.

[10] Diallo FB, Diallo MS, Bangoura S, Diallo AB, Camara Y (1998). Césarienne: facteur de Réduction de Morbidité et de mortalité foeto-maternelle au Centre Hospitalier Universitaire Ignace Deen de Conakry (Guinée). Méd d’Afrique noire.

[11] Häger RM, Daltveit AK, Hofoss D (2004). Complications of cesarean deliveries: rates and risk factors. Am J Obstet Gynecol.

[12] Mariko S La césarienne au Centre de santé de référence de Koutiala: indications et pronostics foeto-maternels. Thèse de médecine 2008.

[13] Nejad SB, Allegranzi B, Syed SB, Ellis B, Pitte D (2011). Infections liées aux soins de santé en Afrique: une étude systématique. Bull OMS.

[14] Lilford RJ, Van Coeverden De Groot HA, Moore PJ, Bingham P (1990). The relative risks of cesarean section (intrapartum and elective) and vaginal delivery: a detailed analysis to exclude the effects of medical disorders and other acute preexisting physiological disturbances. Br J Obstet Gynaecol.

[15] Suwal A, Shrivastava VR, Giri A (2013). Maternal and fetal outcome in elective versus emergency cesarean Section. J Nepal Med Assoc.

[16] Fomulu JN, Tiyou KC, Mbu RE, Nana NP, Leke RJI (2008). Mortalité maternelle à la maternité principale de Yaoundé: étude rétrospective de 2001 à 2006. Health Sci Dis.

[17] Fomulu JN, Tchana TM, Nana NP, Mbu R, Kasia JM (2009). Mortalité maternelle à l'hôpital général de Yaoundé: étude rétrospective sur 5 années (2002-2006). Health Sci Dis.

[18] Racinet CP, Bouzid F (1994). Césariennes: Éditions techniques. Encycl Med Chir (Paris-France), Techniques chirurgicales, Urologie-Gynécologie.

[19] Kemfang Ngowa JD, Ngassam A, Motzebo MR, Kasia JM (2014). Antibioprophylaxie dans les chirurgies gynécologiques et obstétricales propres et propres contaminées à l'Hôpital Général de Yaoundé, Cameroun. Pan Afr Med J.

[20] Jakobi P, Weissman A, Sigler E, Margolis K, Zimmer FZ (1994). Post-cesarean section febrile morbidity: antibiotic prophylaxis in low risk patients. J Reprod Med.

[21] Shrestha S, Shrestha R, Shrestha B, Dongol A (2014). Incidence and risk factors of surgical site infection following cesarean section at dhulikhel hospital. Kathmandu Univ Med J.

[22] Cisse CT, Andriamady C, Faye O, Diouf A, Bouillin D, Diadhou F (1995). Indications et pronostic de l'opération césarienne au CHU de Dakar. Journal de gynécologie obstétrique et biologie de la reproduction.

[23] Deneux-tharaux C, Carmona E, Bouvier-Colle MH, Bréart G (2006). Postpartum maternal mortality and Cesarean delivery. Obstet Gynecol.

